# Gastroenterology services for patients with Cystic Fibrosis across Australia and New Zealand: a multi-stakeholder assessment of patients' and professionals’ perspectives

**DOI:** 10.3389/fped.2023.1322941

**Published:** 2023-12-15

**Authors:** Tamarah E. Katz, Claire E. Wakefield, Christina Signorelli, Andrew S. Day, Angharad Vernon-Roberts, Chee Y. Ooi

**Affiliations:** ^1^ Department of Nutrition and Dietetics, Sydney Children’s Hospital, Randwick, Sydney, NSW, Australia; ^2^ Discipline of Paediatrics & Child Health, Randwick Clinical Campus, School of Clinical Medicine, UNSW Medicine & Health, Sydney, NSW, Australia; ^3^ Kids Cancer Centre, Sydney Children’s Hospital, Randwick, Sydney, NSW, Australia; ^4^ Department of Gastroenterology, Sydney Children’s Hospital, Randwick, Sydney, NSW, Australia; ^5^ Department of Paediatrics, University of Otago Christchurch, Christchurch, New Zealand

**Keywords:** Cystic Fibrosis, gastroenterologist, gastroenterology, survey, Australia, New Zealand, nutrition, service delivery

## Abstract

**Introduction:**

Gastrointestinal (GI) symptoms are common in individuals with Cystic Fibrosis (CF). International research has highlighted that GI care for this group of patients is lacking. Gastroenterology services to CF clinics across Australasia are yet to be examined. This study aimed to describe the current service delivery model and identify areas for improvement that may lead to positive patient outcomes.

**Materials and methods:**

CF clinicians (dietitians, clinical nurse consultants, respiratory consultants), gastroenterologists (GE), and patients or their carers from Australia and New Zealand (NZ) were surveyed online to gather their opinions on CF gastroenterology services provided in their region. Data were analysed using descriptive statistics (frequencies and percentages). Likert scale questions were analysed by grouping responses 1–5 and 6–10, presented alongside the median and interquartile range (IQR). Mann–Whitney *U* and chi-square tests were used to look at differences between stakeholder groups.

**Results:**

One hundred and fifty-six health professionals and 172 patients or their carers completed the survey. Results showed that the current GI model of care is predominantly a publicly funded service delivered outside of CF clinic time. GE are largely not integrated into the CF team and report a lack of training opportunities. There is a higher level of dissatisfaction with the current service model in NZ than Australia.

**Discussion:**

No stakeholder group deemed the current CF gastroenterology service model as adequate, leaving opportunity for transformations in this field. Ideally this study will invigorate the need for promotion and integration of GI services that would ultimately benefit the whole CF community.

## 1. Introduction

Cystic Fibrosis (CF) is the most common lethal genetic disease in the Caucasian population, with a median age of survival of 54.0 years (1). Death is mainly a result of respiratory failure, which correlates closely to nutritional status (2). Malnutrition is not only strongly linked to poorer lung function but it is an independent risk factor for early death (3).

A body mass index (BMI) of greater than or equal to the 50th percentile for age and sex is recommended for patients as it is associated with better forced expiratory volume in 1 s (FEV1) status of 80% predicted or above (4). Achieving this target requires timely and effective management of respiratory exacerbations, optimal treatment of pancreatic insufficiency and ensuring adequate caloric intake. The latter is often difficult to achieve as individuals with CF are commonly affected by GI manifestations such as constipation, pancreatic insufficiency, gastroesophageal reflux, anorexia, loss of taste, and abdominal pain (5, 6). These GI symptoms are often chronic and contribute substantially to morbidity in CF, requiring management by a GE with specialty skills in this area (7, 8). In a recent priority setting exercise in the UK (9), doctors and people with CF ranked GI symptoms as one of the top two most important topics to research. Half of the survey respondents did not feel that the GI needs of people with CF were being met and that the availability of in-depth specialised GI care was lacking. Integration of Gastroenterology and CF care in the hospital setting has been shown to be variable (10), despite a clear need for GE input to address the many intestinal, nutritional, hepatic and pancreatic manifestations of CF (11). The timely recognition, evaluation, longitudinal assessment, and treatment of these GI complications are essential to ameliorate their associated morbidity and mortality (12).

It is important to note that in the present era of highly effective modulators we have witnessed a significant improvement in respiratory morbidity however gastrointestinal gains are far less clear. A 24 week multicentre trial showed a significant decrease across four of five GI domains with ETI therapy (elazecaftor-tezacaftor-ivacaftor) in patients residing in Germany but not the UK, additionally these improvements still did not reach the scores obtained by healthy controls (13). The PROMISE study, a large prospective study from the US, collected data on GI symptoms after 6 months on ETI and found small overall changes (14). It is likely that GI symptomatology will persist and the need for GI care remains as important.

The mean prevalence of CF across Australia is 1.273/10,000 people and in New Zealand (NZ) is 1.048/10,000 people, some of the highest rates in the world (15). However to date, there has not been an examination of gastroenterology services to CF clinics across Australia and NZ (grouped as Australasia). This study aimed to describe the current service delivery model and attitudes towards gastroenterology, and to identify areas for improvement that may lead to positive patient outcomes. Importantly, the goal was to evaluate services from the perspective of those who deliver the services as well as those who receive them, i.e., patients and carers, throughout Australasia.

## 2. Materials and methods

### 2.1. Ethical approval

This study was conducted in Australia in March 2021 by the Randwick CF Clinic, part of the Sydney Children's Hospital Network (SCHN). Ethics was obtained through the SCHN Human Research Ethics Committee (Ref: 2020/ETH02526). The study was conducted in NZ in June 2021 by the Department of Paediatrics, University of Otago, Christchurch. Ethics approval was granted by the University of Otago Human Ethics Committee (Health) (Ref: HD21/027).

### 2.2. Data collection

The study was designed as a cross sectional structured survey completed using online form providers. Three surveys were designed to gather information from three separate stakeholder groups: (1) CF clinicians including dietitians, clinical nurse consultants and respiratory consultants; (2) gastroenterologists (GE), including advanced trainees, working in either or both the public or private domains, seeing paediatric or adult patients with CF or not; and (3) patients diagnosed with CF or their parent/carers. The survey link was distributed via email and social media to the member base of the following groups: Patients/carers with CF: Cystic Fibrosis Australia (CFA), and Cystic Fibrosis NZ (CFNZ). Gastroenterologists: The Gastroenterological Society of Australia (GESA), the New Zealand Society of Gastroenterology (NZSG) and CF Clinicians: special interest groups of Dietitians Australia and Dietitians NZ, special interest groups of Nursing Council of Australia and CF specialist Nurse networks in NZ. The survey link remained open for one month. There were no exclusion criteria, however the survey was only available in English and therefore those from non-English speaking backgrounds would likely have not participated.

### 2.3. Measures

The surveys were formed around the following areas of interest: (i) demographics, (ii) access to gastroenterology services, (iii) outpatient service model, (iv) integration of the GE within the CF team, and (v) training and education opportunities for GE in CF. Questions and response categories were designed to overlap between the three stakeholder groups where possible. The surveys were a mixture of 10 point Likert scale (1 = low level of agreement, 10 = strong agreement), multiple choice, and free text questions (Table 1). The surveys were 23, 31 and 15 questions long for CF clinicians, gastroenterologists and patients/carers respectively.

**Table 1:** Survey questions by stakeholder group.

Survey domain		Stakeholder		
		Patients/carers	CF Clinicians	Gastroenterologists
i	Demographics (Table 2)	Residing state, age of individual with CF^b^ Attending a paediatric/adult (or combined) centre^b^	CF clinician, dietitian or CNC Residing state^b^ Years experience^c^ Working at a paediatric/adult (or combined) centre^b^ Size of centre^b^	GE or advanced trainee^b^ Residing state^b^ Years experience^c^ Working at a paediatric/adult (or combined) centre^b^ Size of centre^b^
ii	Access to gastroenterology services (outpatients) (Figures 1, 2)	Importance of access to gastroenterology services^a^ (scale,1 = not at all important, 10 = extremely important) Reason to see a GE (in the past 12 months)^b^ How often do you need access a GE^b^ (5-item scale, 1 = never, 5 = 4 or more times per year)	Importance of patient access to gastroenterology services^a^ (scale,1 = not at all important, 10 = extremely important) Reason to see a GI^b^ (choose all applicable)	Importance of patient access to gastroenterology services^a^ (scale,1 = not at all important, 10 = extremely important) Reason to see a GE^b^ (choose all applicable)
iii	Outpatient service model (Table)	GE available at hospital, outpatient^a,b^ Familiarity with GE (yes, not sure, N/A)^b^ Wait time^a^ (scale,1 = reasonable time frame, 10 = way too long) Adequacy of service^a^ (scale,1 = not at all adequate, 10 = completely adequate)	Presence of GE on site at hospital^b^ (yes, visiting only, refer to another public service, refer to a private service) Frequency of GE attendance to CF clinic^b^ (5-item scale, 1 = never, 5 = less than once a month) Wait time^a^ (scale,1 = reasonable time frame, 10 = way too long) Adequacy of service^b^ (scale, 1 = not at all adequate, 10 = completely adequate)	How often do you see CF patients in public outpatients^a^ and private clinics^a^ (scale,1 = never, 10 = very often) Frequency of attendance at dedicated CF clinic^b^ (7-item scale, 1 = once a week, 4 = once every 2–3 months, 5 = not invited, 6 = no but invited, 7 = no but used to) Adequacy of service^a^ (scale,1 = not at all adequate, 10 = completely adequate)
iv	Integration of the gastroenterologist within the CF team (Table 2)		Feelings of being supported by CF GE^b^ (yes, some of the time, no, there is no service) Attendance of GE at case conferences^b^ (N/A, no, sometimes, always) Provision of education by GE to team^b^ (yes, no, we don’t have a GE)	Closeness of relationship with CF team^a^ (scale,1 = not at all closely, 10 = very closely) Frequency of attendance to CF case conferences^b^ (4-item scale, 1 = no, 4 = yes, before/after clinics I attend) Frequency of educational presentation to CF team^b^ (4-item scale, 1 = no, 4 = more frequently than annually) Impression of importance of these services to respiratory physicians^a^ (scale,1 = not at all important, 10 = extremely important)
v	Training and Education (Table 2)	Is the GE well informed on CF needs^b^ (Yes always, yes sometimes, no, N/A) Open comments on service^c^	Is the GE well informed on CF needs^a^ (scale,1 = not at all, 10 = always)	Interest in CF^b^ (yes, no, maybe) Frequency of attendance at CF conferences^b^ (4 point scale, 1 = yes, in the past 2 years, 4 = no) Interest in attendance at a CF conference^b^ (yes, yes but only if GI specific content (yes, no, maybe) Interest in attendance at a CF conference focused on GI issues^b^ (yes, no, maybe) Interest in attendance at a course focused in GI issues^b^ (yes, no, maybe) Interest in participating in a program that also included mentorship and other scholarly activities^b^ (yes, no, maybe) Level of knowledge of current gastroenterology/nutrition guidelines^a^ (scale,1 = not at all informed, 10 = very well informed) Adequacy of training^a^ (scale,1 = not at all, 10 = very well trained) Adequacy of training opportunities in Australasia^a^ (scale,1 = not at all adequate, 10 = very adequate) Barriers to specialising in CF^b,c^ Participation in CF research^b^ (yes, no) Open comments on attracting and retaining CF GI^c^

CF, cystic fibrosis; CNC, clinical nurse consultant; GE, gastroenterologist/s; GI, gastrointestinal.

^a^ Likert scale questions used. ^b^Multiple choice questions used. ^c^Free text questions.

### 2.4. Analysis

Data collected from the survey were exported to the Statistical Package for the Social Sciences (SPSS) version 27 (Released 2020; IBM Corp., Armonk, New York, USA) and analysed using descriptive statistics (frequencies and percentages). Likert scale questions were analysed by grouping responses 1–5 and 6–10, presented alongside the median and interquartile range (IQR). Results were analysed in the three stakeholder groups, with data from the two countries pooled. Further analysis of group differences between Australian and NZ health professionals (CF Clinicians and CF Gastroenterologists) was undertaken with Mann–Whitney *U* and chi-square tests where appropriate.

## 3. Results

### 3.1. Demographics

In total, 156 health professionals completed the survey including 102 CF clinicians (*n* = 68 Australian, *n* = 34 NZ) and 54 gastroenterologists (*n* = 40 Australian, *n* = 14 NZ). In addition, 172 patients or their parents/carers completed the survey (*n* = 159 Australia, *n* = 13 NZ). Most of the consumer surveys were completed by a parent of a child (59%) or parent/carer of an adult with CF (10%). The remainder were completed by adults with CF (31%).

### 3.2. Access to gastroenterology services

**Figure 1:**
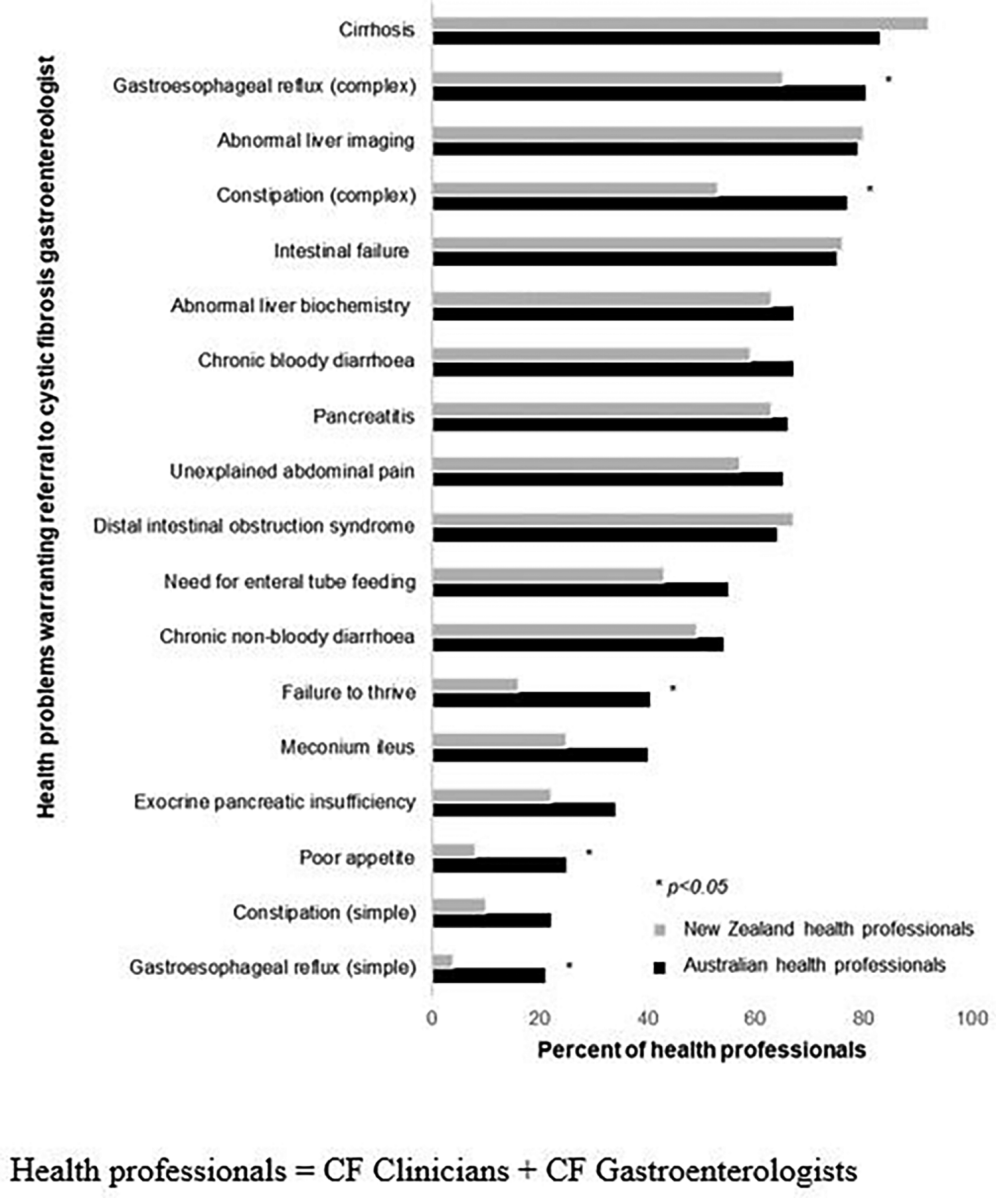
Clinical scenarios that health professionals reported warranted referral to a cystic fibrosis gastroenterologist (Australian *n* = 100; NZ *n* = 49).

All health professionals identified at least one health problem which would warrant referring a patient with CF to a GE (Figure 1). The three most commonly identified health problems were hepatic cirrhosis (88%), abnormal liver imaging (80%), or intestinal failure (76%). The least commonly endorsed reasons to involve a gastroenterologist were for the management of poor appetite (17%), simple constipation (16%), and simple reflux (13%). Other reasons for referral noted by respondents as free text items were liver transplant (*n* = 5) and colon cancer screening (*n* = 4).

A significantly greater proportion of Australian health professionals than NZ health professionals endorsed that the following clinical issues warranted gastroenterology expertise: complex reflux (82% Australia vs. 65% NZ, *p = 0.018*); complex constipation (78% vs. 53%, *p = 0.002*); failure to thrive (41% vs. 16%, *p = 0.003*); poor appetite (25% vs. 8%, *p = 0.014*), and simple reflux (21% vs. 4%, *p = 0.006*). There were no other differences in problems endorsed between Australian and NZ health professionals (all *p > 0.05*).

**Figure 2:**
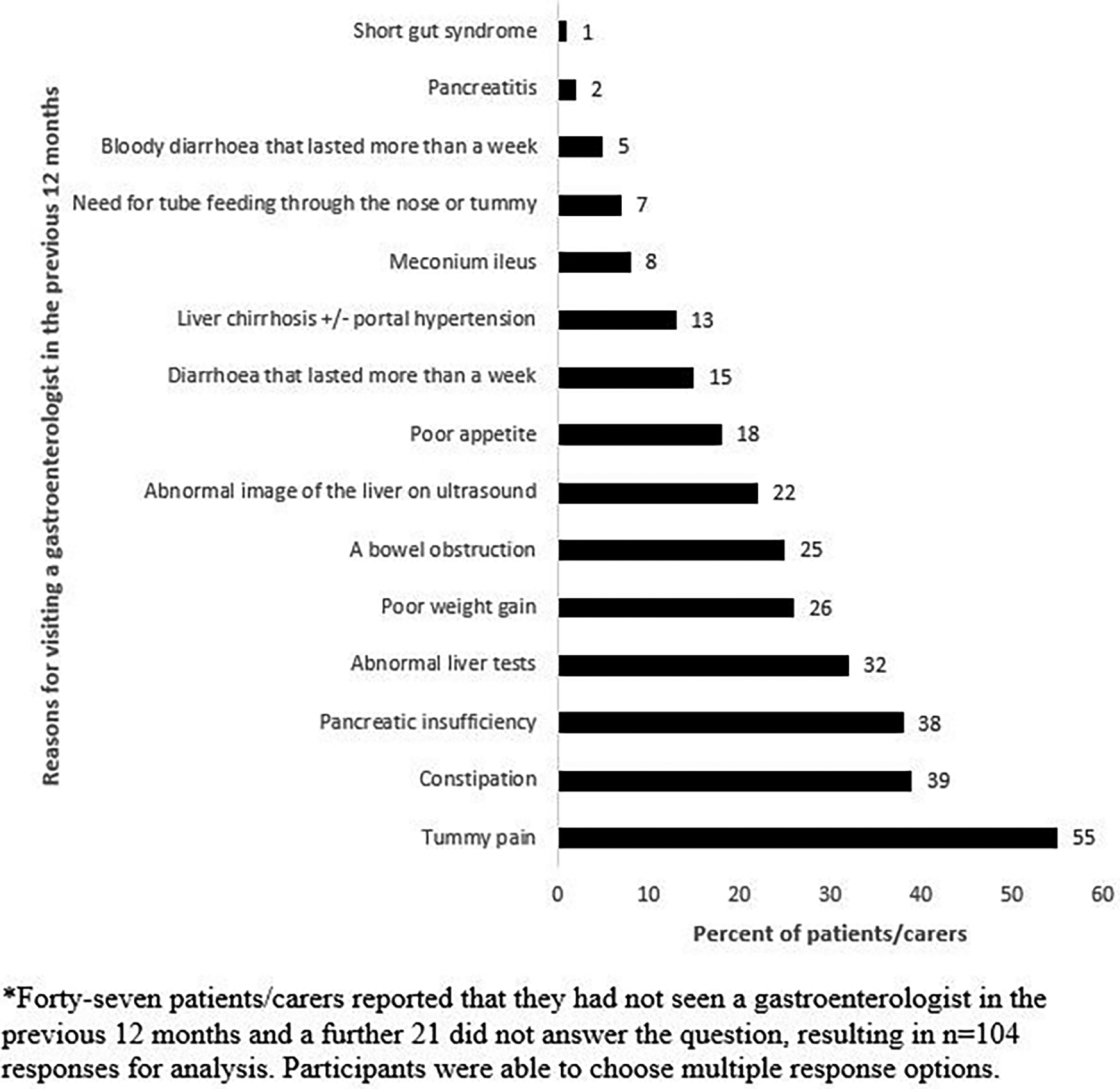
Patient and carer reported reasons for an individual with cystic fibrosis seeing a gastroenterologist in the previous 12 months (*n* = 104)*.

The minority of patients or their carers reported that they had never needed to access a GE: Australian (11%) New Zealand (23%) (Table 2). Among those that had, the three most commonly reported reasons a patient had seen a GE were for abdominal pain (55%), constipation (39%), and for the management of exocrine pancreatic insufficiency (38%) (Figure 2).

**Table 2:** Demographics of health professional and patient/carer survey respondents.

Health Professionals (*N* = 157)			
Characteristic	Aust (*n* = 108) [*N* (%)]	NZ (*n* = 48) [*N* (%)]	Aust and NZ combined data [*N* (%)]
Cystic fibrosis clinicians			
Respiratory doctor	32 (47)	12 (35)	44 (43)
Dietitian	21 (31)	11 (32.5)	33 (32)
Clinical Nurse Consultant/Specialist	15 (22)	11 (32.5)	26 (25)
Gastroenterologists			
Gastroenterologist	30 (75)	14 (100)	44 (81)
Advanced trainees	10 (25)	0	10 (19)
Years of experience, Mean (SD)	12 (9)	13 (10)	
Australian state			
Queensland	34 (31)	—	
Victoria	26 (24)	—	
New South Wales	14 (13)	—	
South Australia	12 (11)	—	
Western Australia	11 (11)	—	
Australian Capital Territory	6 (6)	—	
Tasmania	5 (4)	—	
Northern Territory	0	—	
New Zealand state			
Auckland	—	12 (25)	
Canterbury	—	7 (14)	
Otago	—	6 (13)	
Bay of Plenty	—	4 (8)	
Hawke's Bay	—	4 (8)	
Wellington	—	3 (6)	
Manawatu—Wanganui	—	3 (6)	
Waikato	—	3 (6)	
Northland	—	2 (4)	
Taranaki	—	2 (4)	
Tasman/Nelson	—	2 (4)	
Southland	—	1 (2)	

Patients and Carers (*n* = 172)			
Respondent group,	Aust (*n* = 159) [*N* (%)]	NZ (*n* = 13) [*N* (%)]	Aust and NZ combined data [*N* (%)]
Parent of a child with cystic fibrosis	95 (60)	6 (46)	101 (59)
Parent/carer of an adult with cystic fibrosis	17 10)	1 (8)	18 (10)
Adults with cystic fibrosis	47 (30)	6 (46)	53 (31)
Patient's age (child), median	14 years	10 years	
Australian state,			
New South Wales	41 (26)		
Queensland	34 (21)		
Western Australia	33 (21)		
Victoria	29 (18)		
South Australia	12 (8)		
Australian Capital Territory	2 (1)		
Tasmania	5 (3)		
Northern Territory	3 (2)		
New Zealand province,			
Auckland	—	2 (15)	
Canterbury	—	2 (15)	
Otago	—	1 (8)	
Bay of Plenty	—	1 (8)	
Hawke's Bay	—	0	
Wellington	—	4 (30)	
Manawatu—Wanganui	—	1 (8)	
Waikato	—	0	
Northland	—	1 (8)	
Taranaki	—	1 (8)	
Tasman/Nelson	—	0	
Southland	—	0	
Reported need for gastroenterology review in previous year,			
Never	16 (11)	3 (23)	19 (12)
Once every few years	30 (20)	4 (31)	34 (21)
Once per year	28 (19)	4 (31)	32 (20)
2–4 times per year	57 (38)	1 (8)	58 (36)
4 or more times per year	18 (12)	1 (8)	19 (12)

n, number; SD, standard deviation; Health professionals, CF Clinicians + CF Gastroenterologists; Aust, Australia; NZ, New Zealand.

Numbers and percentages may not add up to 100% due to rounding errors and missing data.

Australian and NZ clinicians and patients/carers alike rated the importance of access to a GE for patients with CF as high (median = 10, IQR: 8–10; median = 10, IQR: 7–10; median of 10; IQR: 7–10) respectively.

### 3.3. Outpatient service model

Ninety percent of Australian CF clinicians reported that they had a GE based at their hospital, with the remainder of responses divided equally between having a visiting GE from another hospital, referring patients to another hospital or to a private GE. Fewer NZ CF clinicians (57%) reported that they had a GE based at their hospital, whilst 37% had visiting GE from another hospital and 6% referred to a private GE.

Fifty-four percent of patients or their carers indicated that they were not familiar with their hospital's GE. Both Australian and NZ GE reported a similar frequency of seeing patients with CF in their public outpatient clinics (median = 5, IQR: 2–9 vs. median = 2, IQR: 2–5; *p* = 0.117), and in their private clinics (median = 2, IQR: 1–3 vs. median = 1, IQR: 1–1; *p* = 0.185).

A small proportion of health professionals reported that a GE attended their CF clinic in both Australia (21%) and NZ (16%) (*p* = 0.497; Supplementary Figure 1). Thirty percent of the non-attending Australian GE indicated that they had previously attended, which was significantly higher than the proportion of NZ GE (4%, *p < 0.001*).

CF clinicians ranked the waiting time to see a GE toward 10 “way too long”, (Australian median = 10, IQR: 8–10; NZ median = 9, IQR: 7–9, *p* = 0.35). On the same scale, patients/carers reported waiting time as a median of 5 (IQR: 3–8). GE indicated that the outpatient service provided at their centre was adequate to meet the needs of patients with CF (Australian median = 7, IQR: 5–8; NZ median = 5, IQR: 4–7). Using the same scale, CF clinicians ranked it lower (Australian median = 5, IQR: 5–9; NZ median = 3, IQR: 2–7). Patients and carers rated their overall satisfaction with their current gastroenterology service as a median score of 5, IQR: 3–8).

### 3.4. Integration of GE within the CF team

Most CF clinicians reported feeling well supported by their hospital gastroenterology services “all” or “some” of the time (Australian 13% and 64%; NZ 39% and 29% respectively). A larger proportion of Australian than NZ clinicians reported that GE sometimes or always attended case conferences when present at clinic (Australia: 67% vs. NZ 26%, *p = 0.003*). Few clinicians reported that their CF team had received any education in the prior 12 months from the GE (Australia: 26%, NZ: 21%).

Most GE perceived that CF specialists feel that gastroenterology services to CF clinics are very important (83% Australian, 71% N*Z*). More Australian GE (63%) reported that they felt they worked closely with the CF team, than NZ GE (29%, *p = 0.03*).

GE reported rarely attending case conferences when present at clinic (31% Australian,14% NZ) or presenting at journal club, educational or other forums (16% Australian, 21% NZ). A minority of GE reported that they were involved in any CF-related research (Australian: 32%, NZ 14%) (Table 3).

**Table 3:** Health professional's perspectives on the integration of gastroenterologists within the cystic fibrosis team and education and training opportunities.

Integration within the cystic fibrosis team				
	Aust CF clin (*n* = 68) [*N* (%)]	NZ CF clin (*n* = 34) [*N* (%)]	*p* value*	Aust & NZ CF clin (*n* = 102) [*N* (%)]
Clinician perceived support of hospital gastroenterology services,				
Well supported all of the time	8 (13)	8 (29)	*p = 0*.*083*	16 (18)
Supported some of the time	38 (63)	11 (39)	* *	49 (56)
Not supported at all	14 (23)	9 (32)	* *	23 (26)
Gastroenterologist attendance at case conferences when present at clinic,				
Attends sometimes or always	28 (67)	14 (74)	***p = 0.003***	42 (69)
Never attends	14 (33)	5 (26)	* *	19 (31)
Clinician perceived knowledge of gastroenterologists of managing CF patients,				
Well informed	39 (71)	11 (34)	***p < 0.001***	50 (57)
Not well informed	16 (29)	21 (66)		37 (43)
Clinician education provided in prior 12 months by gastroenterologist, *n (%)*	13 (26)	3 (21)	*p = 0*.*755*	* *
	Australian gastroenterologists (*n* = 40) [*N* (%)]	NZ gastroenterologists (*n* = 14) [*N* (%)]	*p* value*	Aust & NZ CF clin (*n* = 102) [*N* (%)]
Gastroenterologist perceived value of services to CF clinicians,				
Not very important	6 (17)	4 (29)	*p = 0*.*345*	10 (20)
Very important	30 (83)	10 (71)	* *	40 (80)
Gastroenterologist perceived closeness to CF team, *n* (%)				
Work very closely to CF team	20 (63)	4 (29)	***p = 0.034***	24 (52)
Do not work closely to CF team	12 (37)	10 (71)	* *	22 (48)
Gastroenterologist presentations to CF team, journal club or other forum,				
Presents at least once a year or more	7 (22)	0	*p = 0*.*163*	7 (15)
Rarely presents	5 (16)	3 (21)	* *	8 (17)
Never presents	20 (63)	11 (79)	* *	32 (68)
Gastroenterologist attendance at case conferences presented at clinic,				
Attends sometimes	1 (3)	1 (7)	*p = 0*.*173*	2 (4)
Only if they have a patient to discuss	5 (16)	0	* *	5 (11)
Rarely attends	10 (31)	2 (14)	* *	12 (26)
Never attends	16 (50)	11 (79)	* *	27 (59)
Training and Education Opportunities for Gastroenterologists in Cystic Fibrosis				
	Australian gastroenterologists (*n* = 40) [*N* (%)]	NZ gastroenterologists (*n* = 14) [*N* (%)]	*p value* *	Aust and NZ combined data [*N* (%)]
Gastroenterologist interest in CF, *n* (%)			*p = 0*.*112*	
Interested	16 (55)	3 (21)	* *	19 (44)
Possibly interested	8 (28)	7 (50)	* *	15 (35)
Not interested	5 (17)	4 (29)	* *	9 (21)
Gastroenterologist reported adequacy of training opportunities,				
Adequate training opportunities	13 (45)	2 (14)	***p = 0.049***	15 (35)
Inadequate training opportunities	16 (55)	12 (86)		28 (65)
Gastroenterologist self-perceived training to manage CF patients,				
Adequately trained	23 (79)	4 (29)	***p = 0.001***	27 (63)
Inadequately trained	6 (21%)	10 (71%)		16 (37)
Gastroenterologist self-perceived knowledge on best practice nutrition/gastroenterology CF guidelines,				
Well informed	16 (55%)	2 (14%)	***p = 0.011***	18 (42)
Not well informed	13 (45%)	12 (86%)		25 (58)
Gastroenterologist involved in any CF-related research,	9 (32%)	2 (14%)	*p = 0*.*215*	11 (20)
Gastroenterologist attended CF conference in 2 years prior,	2 (7%)	0	*p = 0*.*314*	2 (4)

n, number; CF, cystic fibrosis.

* Significant differences in bold (*p < 0.05*), according to chi square test. Numbers/percentages may not add up due to rounding errors or missing data.

### 3.5. Training and education opportunities for GE in cystic fibrosis

A greater proportion of Australian CF clinicians (71%) reported perceiving their GE to be well informed on the needs of patients with CF than NZ clinicians (34%, *p < 0.001*) (Table 3). Australian patients/carers reported feeling that their GE was well informed on their needs (median = 7, IQR: 5–9).

Significantly fewer NZ GE reported perceiving their training opportunities to be adequate for the management of individuals with CF than Australian GE (14% vs. 45%, *p = 0.049).* Further, fewer NZ GE felt adequately trained (29% vs. 79%, *p = 0.011*). A greater proportion of Australian CF clinicians reported perceiving their GE to be well informed of best practice guidelines for gastroenterology, nutrition and cystic fibrosis than NZ clinicians (71% vs. 34%, *p = 0.001*) (Table 3).

Most GE were “interested” or “possibly interested” in CF (Australian: 55% “interested”, 28% “possibly interested”; NZ 21% “interested” and 50% “possibly interested”). The most common barrier to wanting to specialise further in CF was a lack of time (Australian 55%, NZ 79%). Not enough training opportunities (38%), not enough clinical exposure to CF (41%) and a lack of funding for CF gastroenterology research (41%) were among the other stated barriers. Other factors reported as free text included: not enough work, not seen as a priority area amongst GE, site specific opportunities based on consultant's interest and trainee's interest, limited hospitals with CF centres, lack of funding for CF gastroenterology services and a lack of support for gastroenterology in general (Table 3).

Few GE (7% Australian, 0% NZ) had attended a CF conference in the preceding two years. GE indicated their preferences for future modes of training and education (Supplementary Figure 2), most popularly the potential to attend a course or conference focused exclusively on gastroenterology issues in CF (48% Australian, 43% NZ*)*.

## 4. Discussion

The key findings arising from this survey-based evaluation include: divergent views on the importance of unexplained abdominal pain by CF health professionals and persons with CF; lack of collaboration between CF GE and the CF multidisciplinary team (MDT); inadequate training opportunities for CF GE and a higher level of dissatisfaction with the current CF gastroenterology model of care in NZ than Australia. The following discussion elaborates on these points.

Unexplained abdominal pain was the most nominated reason for a patient with CF or their carer to require GI services in the previous year. In contrast, health professionals ranked it as ninth on a list of clinical scenarios that would warrant referral to a GE. Pain in the abdomen, chest and head are the areas with the highest frequency for those with CF (16). Lusman et al. (17) highlights that abdominal pain in CF can be challenging for the clinician and patient alike as the differential diagnosis can be complex. Importantly, among the group with CF, pain is associated with lower quality of life along with more anxiety and depression, worse physical function, impairment of sleep, and restriction of activities and work (16, 18). A paediatric study has shown that children with CF have worse GI quality of life compared to healthy controls, specifically in the domains of abdominal pain, constipation, diarrhoea, gas/bloating, and worry regarding stomach ache (19). The results shown in this study may indicate that the high GI symptom burden among patients with CF may be underestimated and/or not discussed with their clinicians. GI symptom burden has been shown to be associated with dissatisfaction with GI targeted treatments among those with CF, thereby indicating a need for increased attention to this symptomatology (20).

On surveying GE, it was clear that the majority perceived that their services were viewed as important by respiratory physicians, but the minority of CF Clinicians felt well supported by their CF GE. Cohesion between the specialities may be hampered by limited opportunities to meet in person with the majority of GE not present at case conference discussions, weekly education sessions or at CF clinic. In NZ this may be especially difficult with the presence of an onsite GE reported in this survey as only 57%. This finding has also been reported in a previous survey whereby only 22% of centres in the UK had access to face-to-face inpatient review by a GE for patients with CF, and only 26% had a named GE to whom they referred patients (10). Interestingly 32% of Australian and 64% of NZ GE reported that they have never been invited to attend clinic. This is perhaps a missed opportunity for a patient-centric approach to care and may stem from a presumption by CF Clinicians that CF GE will not or cannot attend.

It is clear that there are poor training opportunities for CF GE, poor engagement with existing education events such as CF conferences and lack of participation in CF GI research. This raises concerns for the future of CF GE and the expertise needed to manage the next generation of individuals with CF. This should also be considered in the context of current ETI therapy which does not appear to ameliorate GI symptoms further cementing the importance of the CF GE (13, 14). There is a lot of potential interest in CF from GE but time, funding and clinical exposure remain significant, albeit, modifiable factors. In a recent UK survey CF Clinicians identified lack of time, funding as well as challenges related to clinic capacity and infection control as perceived barriers to CF gastroenterology (10). GE in Australia (17%) and NZ (21%) reported that they were not interested in CF, however, the survey did not explore reasons for this which would be useful information to assist in engaging future trainees. Previous research has reported that improvements to gastroenterology services for patients with CF may be achieved by increasing CF interest and expertise of GE, as well as coordinating joint working practices in the form of CF gastroenterology clinics, MDT's, and teaching (10). It is clear that improvements need to be made to coordinate clinical and education endeavours between the two specialities.

This survey unexpectedly found that CF Clinicians and GE working in NZ had a greater level of dissatisfaction with the CF gastroenterology service model compared with those in Australia, ranking the service as 3 and 5 out of 10 respectively. NZ GE reported that they did not work closely with the CF team. Furthermore they rated the adequacy of training opportunities, the adequacy of their personal training and how well informed they were on CF matters as significantly less than their Australian counterparts. The current study was unable to ascertain the reason for this disparity. However, potential causes may include smaller size of CF units and geographical variations in gastroenterology staffing.

Underpinning the aforementioned issues is the obvious lack of CF GI guidelines across Australasia. Guidelines exist for general CF care (21, 22), nutrition (23), physiotherapy (24) and diabetes (25). Previous work has shown that in the absence of clinical guidelines there will be variation in management of CF manifestations (26).

### 4.1. Study limitations

The percent of patients captured, calculated using the most recently available CF Data Registry reports were low: Australia (4%) and New Zealand (2.6%) (27, 28). It was not possible to calculate the capture rate for CF GE and CF Clinicians as this data isn't available. It was also not possible to calculate response rates for patients, CF GE or CF Clinicians given the recruitment methods included posting on social media. Our survey design may have been subject to recall bias, it is also possible that questions were interpreted differently within the group. Respondents may have had a vested interest in CF gastroenterology issues and as our survey was only available in English, consumers from diverse backgrounds may have been underrepresented. Further research may be warranted to understand potential differences in the needs or preferences of this priority group. Finally, all GE were invited to participate in this study, whether they actively see individuals with CF or not. It is fair to say that those who do may be better placed to respond to some of the survey questions. This study provides valuable, first insights into the CF gastroenterology service model in Australasia, additionally it explores common CF GI issues experienced by children and adults, which can help inform future studies.

## 5. Conclusion

No stakeholder group deemed the current gastroenterology outpatient service model as entirely adequate, leaving opportunity for meaningful transformations in this field. Ideally the data from this study will invigorate the need for promotion and integration of gastroenterology services that would ultimately benefit the whole CF community.

## Data Availability

The original contributions presented in the study are included in the article/**Supplementary Material**, further inquiries can be directed to the corresponding author.
