# Supplementary material for: Gastroenterology services for patients with Cystic Fibrosis across Australia and New Zealand: a multi-stakeholder assessment of patients' and professionals’ perspectives
Source: Front Pediatr. 2023 Dec 15;11:1322941. doi: 10.3389/fped.2023.1322941 (PMC10755025; doi:10.3389/fped.2023.1322941)
Supplement: Supplementary file 1 [file Table1.docx]

Supplementary Figure 1: Frequency of GI attendance at CF outpatient clinics (reported as % of respondents: Australian health professionals (n=95) and New Zealand health professionals clinician (n=49)

**
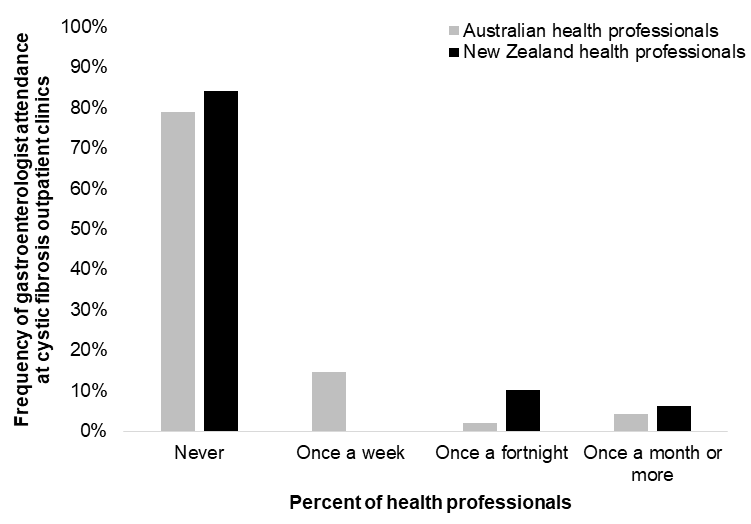
**

Health professionals = CF Clinicians + CF Gastroenterologists
