# Supplementary material for: Gastroenterology services for patients with Cystic Fibrosis across Australia and New Zealand: a multi-stakeholder assessment of patients' and professionals’ perspectives
Source: Front Pediatr. 2023 Dec 15;11:1322941. doi: 10.3389/fped.2023.1322941 (PMC10755025; doi:10.3389/fped.2023.1322941)

Supplementary Figure 2. Gastroenterologists interest in various potential training and educational opportunities (reported as % of respondents: Australian gastroenterologists (n=29) and New Zealand gastroenterologists (n=14)


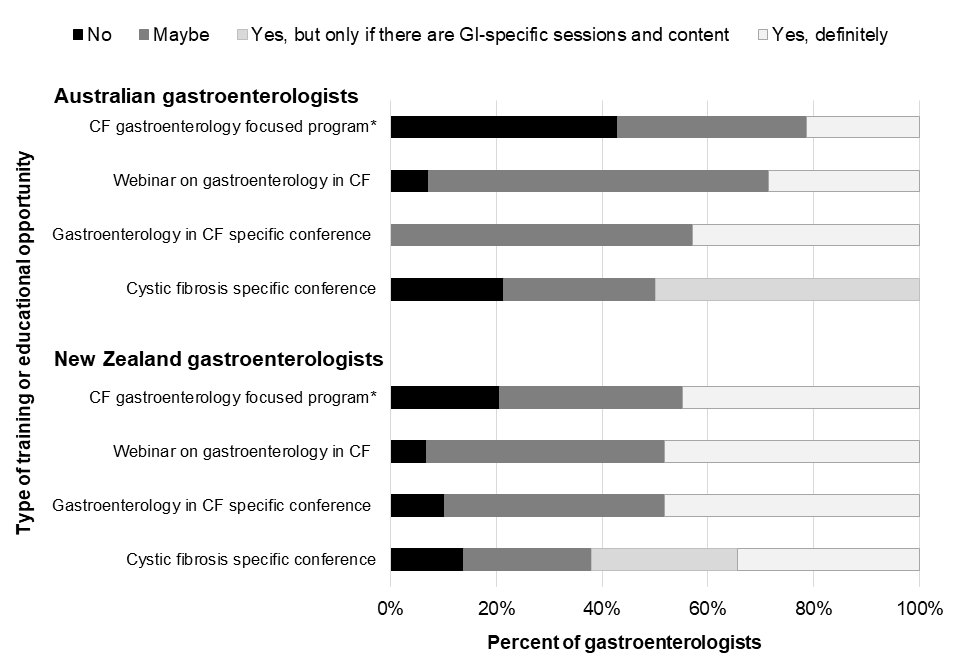

Supplement: Supplementary file 2 [file Table2.docx]
